# Large Animal Models for Simulating Physiology of Transfusion of Red Cell Concentrates—A Scoping Review of The Literature

**DOI:** 10.3390/medicina58121735

**Published:** 2022-11-27

**Authors:** Melanie Berndt, Maximilian Buttenberg, Jan A. Graw

**Affiliations:** 1 Department of Anesthesiology and Operative Intensive Care Medicine (CCM, CVK), Charité–Universitätsmedizin Berlin, 13353 Berlin, Germany; 2 Department of Anesthesiology and Intensive Care Medicine, Ulm University, 89081 Ulm, Germany

**Keywords:** blood transfusion, red cell concentrates, erythrocyte storage, large animal model, transfusion model

## Abstract

*Background and Objectives*: Transfusion of red cell concentrates is a key component of medical therapy. To investigate the complex transfusion-associated biochemical and physiological processes as well as potential risks for human recipients, animal models are of particular importance. This scoping review summarizes existing large animal transfusion models for their ability to model the physiology associated with the storage of erythrocyte concentrates. *Materials and Methods*: The electronic databases PubMed, EMBASE, and Web of Science were systematically searched for original studies providing information on the intravenous application of erythrocyte concentrates in porcine, ovine, and canine animal models. *Results*: A total of 36 studies were included in the analysis. The majority of porcine studies evaluated hemorrhagic shock conditions. Pig models showed high physiological similarities with regard to red cell physiology during early storage. Ovine and canine studies were found to model typical aspects of human red cell storage at 42 days. Only four studies provided data on 24 h in vivo survival of red cells. *Conclusions*: While ovine and canine models can mimic typical human erythrocyte storage for up to 42 days, porcine models stand out for reliably simulating double-hit pathologies such as hemorrhagic shock. Large animal models remain an important area of translational research since they have an impact on testing new pharmacological or biophysical interventions to attenuate storage-related adverse effects and allow, in a controlled environment, to study background and interventions in dynamic and severe disease conditions.

## 1. Introduction

### 1.1. Transfusion Therapy and Red Blood Cell Storage Lesion

The transfusion of red cell concentrates is a common cross-disciplinary medical intervention and often a key component of therapy in a variety of clinical settings. Despite major improvements in the preservation of erythrocyte concentrates that allow storage for up to 42 days, adverse storage effects and their potentially harmful impact on recipients are a highly controversial topic [1]. These complex storage-related biochemical changes, commonly referred to as “storage lesions”, are characterized by an irreversible oxidation of proteins, a loss of membrane phospholipids, a rapid depletion of 2,3-diphosphoglycerate, and a gradual reduction of adenosine triphosphate. These biochemical alterations modify red blood cell morphology at the expense of deformability and ultimately lead to decreased oxygen transport [2,3]. In addition, the altered red cell plasticity renders erythrocytes prone to hemolysis with concomitant release of potassium, iron, lactate dehydrogenase, microparticles, heme, and hemoglobin [4,5,6]. These structural changes influence the adhesion and interaction of erythrocytes with endothelial cells and consequently compromise microvascular flow and trigger immune reactions. This is mainly caused by an increased release of bioactive microvesicles and inflammatory cytokines, which in turn promotes systemic organ injury, particularly in vulnerable patient groups [3,4].

### 1.2. Importance of Animal Models in Biomedical Research

Although in vitro studies have adequately demonstrated storage-related physiological and biochemical alterations, the transferability of observed changes and potentially harmful sequelae upon transfusion of red cell concentrates need to be further investigated in vivo [2,7]. Animal models are still of particular importance to gain a better understanding of these complex biochemical and pathophysiological processes and are essential for the evaluation of the safety and efficacy of different therapies [8]. However, the use of animals in research must be approached and regulated with the highest ethical standards and can only be justified when harm is reduced to a minimum. A crucial ethical principle, the “3R principle”, was first presented by William Russell and Rex Burch in 1959 and is now embedded in many national and international guidelines [9,10]. Animal studies may only be performed if the scientific aim cannot be accomplished by other methods (replacement), the number of animals used must be reduced to the minimum possible number (reduction), and conditions must be applied to better animal welfare (refinement) [9,10].

Porcine models are increasingly gaining favor over rodent models in translational research due to their similarity to humans in both their anatomy and their metabolic profile [8,9]. Ovine and canine animal models are of particular importance for respiratory and cardiovascular pathologies [11,12,13]. With regard to transfusion medicine, several large animal studies have contributed to current clinical treatment strategies and recommendations because large animal studies allow the investigation of research questions that cannot be explored in humans because of accompanying dynamics and risks, such as in severe shock or severe anemia. For example, several recent large animal studies have contributed to actual transfusion strategies in the military setting or provided the background knowledge for recommendations on the risks of iron supplementation compared with transfusion of packed red blood cells in several clinical conditions [14,15,16,17,18]. Therefore, it is of major importance to comprehensively evaluate the potential of these particular animal models to simulate the pathophysiology of red blood cell transfusion as well as the possible pitfalls of their use in modeling the physiology and biochemistry associated with prolonged storage of red cell concentrates.

Since the translation of transfusion-associated research questions to appropriate animal models is key to understanding physiology and to developing future therapeutic approaches for human health, the aim of the current study is to systematically screen and analyze the existing literature on transfusion therapy in major large animal models (porcine, ovine, and canine) in vivo and evaluate their ability to adequately model physiology and biochemistry associated with prolonged storage of red cell concentrates.

## 2. Material and Methods

### 2.1. Search Strategy

A comprehensive search of the literature was conducted to identify all studies providing information on the use of red blood cell concentrates in porcine, ovine, and canine animal models. The databases PubMed (Public Medicine), EMBASE (Excerpta Medica database) and Web of Science were systematically searched without any restrictions using Ovid (Ovid Technologies) on 15 November 2021. Results were obtained by applying the following search strategy to the different databases: [(rbc transfusion *) OR (red blood cell transfusion *) OR (rcc transfusion *) OR (red cell concentrate * transfusion *) OR (erythrocyte transfusion *) OR (packed red blood cells transfusion *)] AND [(animal model)].

This search syntax was slightly modified for the use in EMBASE as follows in order to guarantee maximal comparability of search results: (1) “rbc transfusion” OR “red blood cell transfusion” OR “rcc transfusion” OR “red cell concentrate transfusion” OR “erythrocyte transfusion” OR “packed red blood cells transfusion” (2) #1 AND “animal model”.

### 2.2. Inclusion and Exclusion Criteria

**Figure 1:**
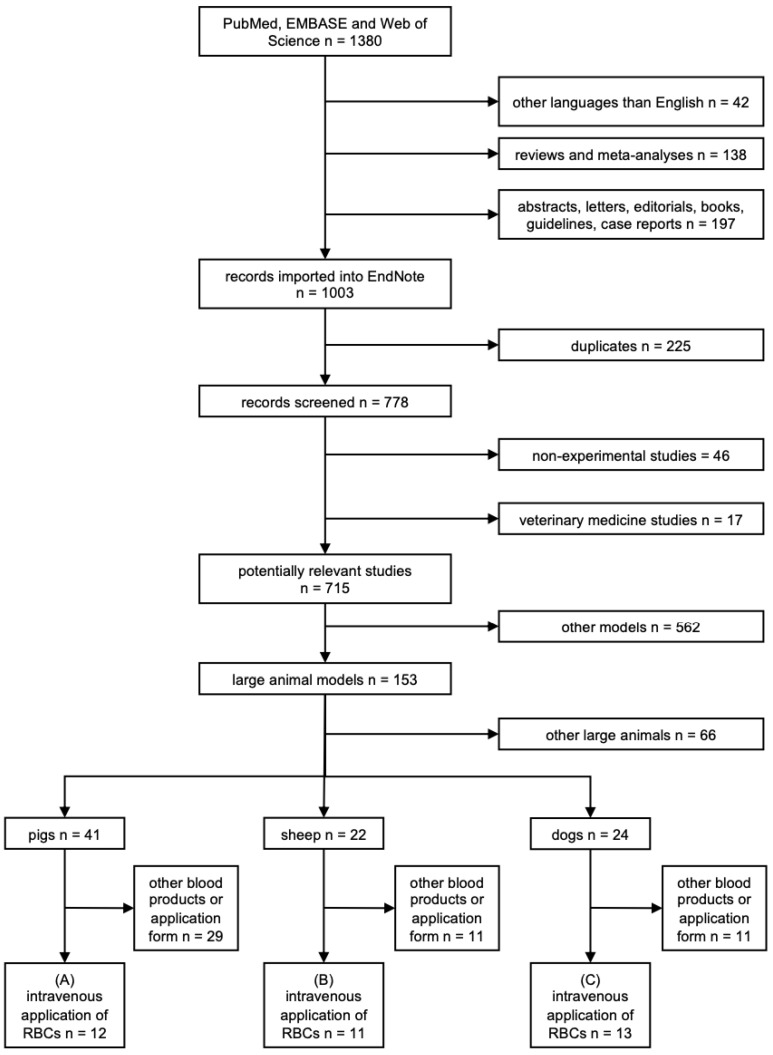
Study flow diagram. PubMed: Public Medicine, EMBASE: Excerpta Medica dataBASE, RBCs: red blood cell concentrates.

To be eligible for inclusion, all identified publications had to be experimental studies of pigs, sheep, or dogs that had received an intravenous transfusion of erythrocyte concentrates. Only studies published in English were considered eligible for inclusion. Figure 1 illustrates inclusion and exclusion criteria applied in a hierarchical manner: (1) All non-English papers, reviews, and meta-analyses, as well as conference abstracts, letters, editorials, book chapters, guidelines and case reports, were excluded. (2) Searches were imported to the reference management software EndNote 20 (Thomas Reuters, CA), by which duplicates were identified and deleted. The remaining copies were detected and deleted manually. In addition, veterinary medicine studies, as well as non-experimental studies not already excluded in step 1, were eliminated from further analysis. (3) All studies using models other than large animal models (for example rodent models) were discarded. (4) To ensure comparability within an animal species, large animal models with n < 8 transfusion-related publications, such as studies on rabbits, hamsters, or non-human primates, were excluded. Therefore, only studies on pigs, sheep, or dogs remained for analysis. (5) Ultimately, remaining experimental studies without transfusion of any porcine, ovine, or canine red cell concentrates or a non-intravenous route of transfusion–such as intraosseous infusion–were eliminated. This resulted in 12 publications with information on erythrocyte transfusion in pigs, 11 publications on erythrocyte transfusion in sheep, and 13 publications on erythrocyte transfusion in dogs.

All decisions on eligibility for inclusion were made by screening the titles and abstracts of the studies. The full text was analyzed if the information obtained from the abstract was not sufficient for decision-making. In case of discrepancy, potential studies were evaluated by two anesthesiologists experienced in small and large animal research on transfusion models. A final decision was made by a majority vote.

### 2.3. Data Extraction

The following information was extracted from studies meeting the inclusion criteria: characteristics of the study population (group sizes, strain of animal, gender, age, weight, and type of model), documentation of drop-outs and the main objective of the study as well as information on the preparation of erythrocytes (number of donor animals and centrifugation) and their storage (anticoagulant, additive solution, temperature, and time). Furthermore, for the evaluation of storage-related changes of packed red blood cells, data on 24 h in vivo red blood cell survival, 2,3-diphosphoglycerate, adenosine triphosphate, hemoglobin, free hemoglobin, and hematocrit levels, as well as on hemolysis index [(free Hb * hematocrit)/donor unit Hb] were extracted. If required, hemoglobin concentrations were converted to g/dL and data on measurements of free hemoglobin were converted to mg/dl. Data not provided in the full text of the publication were estimated from the enclosed figures.

### 2.4. Assessment of Quality of Study

The quality of eligible studies was assessed according to current guidelines for reporting scoping reviews using PRISMA [19]. The review was formally registered: 10.17605/OSF.IO/R26US.

## 3. Results

### 3.1. Search Results

The search identified a total of 1380 publications, with 1368 studies discarded for not meeting the inclusion criteria Figure 1. Of 715 non-duplicated studies, 153 experimental studies used a large animal model. Of these publications, 41 studies were performed on pigs, 22 on sheep, and 24 on dogs. Further full-text reviewing resulted in a total of 36 studies that transfused red blood cell concentrates intravenously. These 36 studies, representing a total number of 320 pigs (A), 483 sheep (B), and 440 dogs (C), were included in the final analysis Figure 1.

### 3.2. Basic Study Characteristics

Table 1 summarizes the characteristics of the study population, drop-outs, as well as the main objective of the 12 publications on pigs (A). Table 2 outlines data on the 11 studies in sheep (B) and Table 3 on the 13 studies in dogs (C).

(A) In 12 out of 36 studies, a total of 320 pigs were included, with the number of animals ranging from five to 60 per study [15,20,21,22,23,24,25,26,27,28,29,30]. With a majority of six studies, transfusion of red cell concentrates was most frequently carried out in female swine [15,22,25,26,29,30]. Four studies, however, did not provide any data on the gender of the animals [20,21,27,28]. Only two papers investigating the effects of different volume replacements in asphyxia and hemorrhagic shock model of 32 h old newborn piglets reported data on age [20,21]. In nine out of 12 studies, animals received erythrocyte concentrates without the combination of other blood products [15,20,21,22,23,24,25,26,30]. Fresh frozen plasma or lyophilized plasma, however, was applied in a ratio of 1:1 with packed red blood cells in the remaining three studies [27,28,29]. Of the included publications, three studies reported an application of porcine erythrocytes as a top-load transfusion in healthy swine [22,24,25]. With a total of eight out of 12 studies, transfusion was carried out most frequently in a model of hemorrhagic shock [15,20,21,23,27,28,29,30].
(B) Eleven of the 36 included publications, with a total of 478 sheep, reported a similar heterogeneity in group sizes varying from 10 to 253 animals per study [31,32,33,34,35,36,37,38,39,40,41]. Only two studies of the ones that provided data on the gender of animals used male sheep (34, 35). Six out of 8 studies reported data on animal age, and sheep were most frequently older than three months [31,32,34,35,36,37]. Only two publications stated the use of much younger animals [40,41]. In 5 out of the 11 studies, ovine erythrocytes were transfused as a top-load model [31,33,34,37,38]. Other transfusion models, such as hemorrhagic shock or anemia, were comparatively less frequently used [35,36,39,40,41]. All 11 studies applied ovine red blood cell concentrates without the combination of other blood products [31,32,33,34,35,36,37,38,39,40,41]. However, the study by Muenster et al. exposed erythrocyte concentrates to oxygen or nitric oxide prior to transfusion [21].
(C) In 13 out of the 36 included studies, a total of 440 dogs were included, with only three studies describing a repeated use of the same animals [14,42,43,44,45,46,47,48,49,50,51,52,53]. Study populations showed similar ranges compared to the other two species, with studies using six to 76 dogs per study [14,42,43,44,45,46,47,48,49,50,51,52,53]. In three out of 13 publications, male and female dogs were included in the same study [42,50,53]. For all studies in pigs and sheep, animals of only one sex were used for each study. However, the studies utilizing canine models were markedly dominated by male dogs [42,50,51,52,53]. Beagles were preferably chosen as a breed, with seven publications providing data in this regard [14,43,44,45,46,47,48]. Reported age mostly varied between 1 and 2.5 years, with only three studies deviating from this range [14,42,43,44,45,46,47,48,49,50,53]. Of the included studies, six publications reported the transfusion of canine red blood cell concentrates in a model of pneumonia [14,43,45,46,47,48]. Furthermore, three studies described the use of a hemorrhagic shock model [44,51,52]. However, two papers reported an application of erythrocytes in a top-load transfusion model, and none of the reviewed studies used healthy dogs [14,42,43,44,45,46,47,48,49,50,51,52,53].

**Table 1:** Characteristics of included porcine studies (group A), sorted by year of publication.

Author [Reference] [Year]	Place of Study	Control Group [Number]	Intervention Group [Number]	Strain of Animal	Gender	Weight (kg)	Type of Model	Objective of Study	Drop-Outs	
									No. of Animals [Group]	Reason
Weber et al. [20] [2019]	GER	Sham [6]	RBC [9] 0.9% saline [6]	N/A	N/A	1.220 [1.060–1.495]	Asphyxia/hemorrhagic shock	Effects of different volume replacements on lung injury	N/A	N/A
Weber et al. [21] [2019]	GER	Sham [6]	RBC [9] 0.9% saline [6]	N/A	N/A	1.220 [1.060–1.495]	Asphyxia/hemorrhagic shock	Inflammatory and cardiac consequences of neonatal AH	N/A	N/A
Wozniak et al. [22] [2018]	UK	Sham [6]	D14 RBC [10] D14 Wash. RBC [6] D14 Rej. RBC [10]	LW × LR	female	50–70	RBC transfusion	Effects of washing/rejuvenation of RBCs on storage lesion associated kidney and lung injury	4 [2 D14 RBC; 2 D14 Rej. RBC]	Refractory hypoxemia or cardiovascular instability
Biagini et al. [23] [2018]	BRA	RL [8]	D14 RBC [8]	Agroceres^®^ pigs	male	68 [± 3.3]	Hemorrhagic shock	Effects of RBC transfusion on cardiopulmonary function and inflammation	N/A	N/A
Watts et al. [15] [2015]	UK	0.9% saline [9]	RBC:FFP [9] RBC [6]	LW × LR	female	43–56	Polytrauma /hemorrhagic shock	Prehospital resuscitation with blood products as a possibility to avoid ATC	N/A	N/A
Biagini et al. [24] [2014]	BRA	N/A	RBC [5]	Agroceres^®^ pigs	male	37–38 ^a^ 60 ^b^	RBC transfusion	Viability of swine RBCs stored for 14 days	1 [N/A]	Hypoxia
Patel et al. [25] [2013]	UK	Sham [7]	D14 RBC [6] D42 RBC [7]	FLW × LR	female	50–70	RBC transfusion	Transfusion of allogeneic RBC as a cause of PD Effects of RBC storage duration on severity of PD	4 [2 D14; 2 CPB + D42]	Cardiovascular instability or refractory hypoxemia
			CPB [7] CPB + D42 [9]				Post-cardiac surgery acute lung injury	Effects of RBC transfusion on pulmonary function and interaction with CPB		
Patel et al. [26] [2011]	UK	Sham [9]	CPB [7] Sham + RBC [8] CPB + RBC [7]	FLW × LR	female	50–70	Post-cardiac surgery acute kidney injury	Effects of anemia therapy with allogenic RBC transfusion during CPB	N/A	N/A
Spoerke et al. [27] [2010]	USA	N/A	FFP [8] LP [8] FFP:RBC [8] LP:RBC [8]	Yorkshire crossbred swine	N/A	N/A	Polytrauma /hemorrhagic shock	Effect of RBCs on clotting parameters	N/A	N/A
Spoerke et al. [28] [2009]	USA	FFP [8]	LP [8] FFP:RBC [8] LP:RBC [8]	Yorkshire crossbred swine	N/A	N/A	Polytrauma /hemorrhagic shock	Effects of resuscitation with LP on clotting factor activity and coagulopathy correction	N/A	N/A
Alam et al. [29] [2009]	USA	Sham [6]	FWB [14] Hextend [14] FFP:RBC [13] FFP [13]	Yorkshire swine	female	40 [±5]	Polytrauma /hemorrhagic shock	Resuscitation with blood components as a possibility to reverse coagulopathy	N/A	N/A
Buchholz et al. [30] [1999]	USA	CPDA-1 RBC [5]	Adsol RBC [5]	Pitman-Moore mini-pigs	female	90–133	Hemorrhagic shock	Effects of massive infusion of Adsol and CPDA-1 on metabolism and circulation	2 [CDPA-1]	Cardiorespiratory arrest

^a^: homologous animal; ^b^: autologous animal. Abbreviations: GER: Germany; UK: United Kingdom; BRA: Brasil; USA: United States of America; RBC: red blood cell concentrates; D14 RBC: 14-day-old stored porcine red cell units; D42 RBC: 42-day-old 14-day-old stored porcine red cell units; D14 Wash. RBC: washed 14-day-old stored porcine red cell units; D14 Rej. RBC: rejuvenated 14-day-old stored porcine red cell units; RL: Ringer’s lactate solution; FFP: fresh frozen plasma; FWB: fresh whole blood; LP: lyophilized plasma; CPB: cardiopulmonary bypass; CPDA-1: citrate-phosphate-dextrose-adenine-1 solution; Adsol: adenine, dextrose, sorbitol, sodium chloride and mannitol solution; AH: asphyxia; PD: pulmonary dysfunction; LWxLR: Large-White-Landrace crossbred pigs; FLWxLR: Farm-bred Large-White-Landrace crossbred pigs.

**Table 2:** Characteristics of included ovine studies (group B), sorted by year of publication.

Author [Reference] [Year]	Place of Study	Control Group [Number]	Intervention Group [Number]		Strain of Animal	Gender	Age (Months)	Weight (kg)	Type of Model	Objective of Study	Drop-Outs	
											No. of Animals [Group]	Reason
Muenster et al. [31] [2016]	USA	FRBC + O2 [4] FRBC + NO [8]	SRBC + O2 [8] SRBC + NO [9] Washed SRBC [5]		Polypay	N/A	3–4	33 [±2]	RBC transfusion	Effects of NO treatment and washing of RBCs before transfusion	N/A	N/A
McDonald et al. [32] [2015]	AUS	Sham [4] S-ALI [7] ECMO [7]	S-ALI + ECMO [8] S-ALI + ECMO + RBC [14]		Border Leicester-SAMM cross	N/A	12–36	48.6 [±6]	S-ALI/ECMO	Effects of S-ALI, ECMO and RBC transfusion on oxidative stress and plasma selenium levels	N/A	N/A
McCutcheon et al. [33] [2015]	UK	RBC [7]	P-CAPT RBC [7]		ARQ/ ARQ PrP	N/A	N/A	N/A	RBC transfusion	Efficacy of the P-CAPT prion removal filter	1 [RBC]	N/A
Simonova et al. [34] [2014]	AUS	N/A	D5 RBC [6] D38 RBC [6]		Merino	male	12–36	40.7 [±1.7]	RBC transfusion	Development of an ovine RBC transfusion model in comparison to humans	N/A	N/A
Fung et al. [35] [2013]	AUS	0.9% saline [2] HA 4% [2]	D5 RBC [5] D38 RBC [5]		Merino	male	12–36	40.2 [±2.9]	Hemorrhagic shock	Comparison of fresh versus old RBC transfusion	N/A	N/A
Baron et al. [36] [2013]	USA	WB [6]	RBC [6] RBC + NO [5]		Polypay	N/A	3–4	28–35	Hemorrhagic shock	Adverse effects of RBC transfusion after prolonged storage and possible prevention by NO inhalation	N/A	N/A
Lacroux et al. [37] [2012]	FRA	Sham [5]	WB [5] RBC [5] Plasma [5] Buffy-Coat [5] RBC LD [5] Plasma LD [5] RBC LD/PR1 [5] RBC LD/PR2 [5]		VRQ/ VRQ	N/A	6–10	N/A	RBC transfusion	Potential of blood products to transmit scrapie through transfusion route and the efficacy of LD and LD/PR filters in risk reduction	N/A	N/A
McCutcheon et al. [38] [2011]	UK	WB [9]	WB [8] ^a^ RBC [9] ^a^ Plasma [9] ^a^ Buffy-Coat [8] ^a^ Platelets [9] ^a^	RBC [28] ^b^ Plasma [29] ^b^ Buffy-Coat [29] ^b^ Platelets [28] ^b^ RBC LD [29] ^b^ Plasma LD [29] ^b^ Platelets LD [29] ^b^	ARQ/ ARQ PrP	N/A	N/A	N/A	RBC transfusion	Risk of different blood components to transmit CJD via transfusion	N/A	N/A
Jonker et al. [39] [2011]	USA	D10 Sham [5]	D10 Anemia [6] D20 Sham [7] D20 Anemia + RBC [7]		Mixed western	N/A	109–129 GA	2.4 ^c,^* 3.7 ^d,^*	Anemia	Effects of RBC transfusion on the growth and proliferation of cardiomyocytes	N/A	N/A
Vane et al. [40] [2002]	USA	RL [6]	DCLHb [6] RBC [7]		Merino	N/A	N/A	31.1 [±1.2]	Anemia	Comparison of the systemic effects of DCLHb and RBC transfusion	2 [DCLHb]	Hemodynamic instability or dysfunctional hindlimbs
Widness et al. [41] [2000]	USA	N/A	RBC [10]		Mixed Dorset and Suffolk	N/A	5.7 [±0.2] days	5.74 [±0.25]	Anemia	Cardiovascular and metabolic responses after RBC transfusion	N/A	N/A

^a^: donors received 9 inoculations with 5 g of BSE brain homogenate; ^b^: donors received 30 inoculations with 5 g of BSE brain homogenate; ^c^: 119 days GA; ^d^: 129 days GA; *: estimated value according to published figure; Abbreviations: USA: United States of America; UK: United Kingdom; AUS: Australia; FRA: France; RBC: red blood cell concentrates; FRBC: fresh red blood cell concentrates; SRBC: stored red blood cell concentrates; NO: nitric oxide; S-ALI = smoke induced acute lung injury; ECMO = extracorporeal membrane oxygenation; P-CAPT = prion removal P-CAPT filter; D5: stored for 5 days or less; D38: stored for 35–42 days; HA 4%: human albumin 4% (Albumex^®^); WB: whole blood; LD: leuco-depleted; PR1: prion reduction manufacturer 1; PR2 = prion reduction manufacturer 2; CJD: Creutzfeldt-Jakob disease; D10: killed after 10 days of study; D20: killed after 20 days of study; GA: gestational age; RL: Ringer’s lactate solution; DCLHb: diaspirin cross-linked hemoglobin.

**Table 3:** Characteristics of included canine studies (group C), sorted by year of publication.

Author [Reference] [Year]	Place of Study	Control Group [Number]	Intervention Group [Number]	Strain of Animal	Gender [Number]	Age (Years)	Weight (kg)	Type of Model	Objective of Study	Drop-Outs	
										No. of Animals [Group]	Reason [Number]
Callan et al. [42] [2021]	USA	AIHA FRBC [20] ITP FRBC [1]	AIHA SRBC [18] ITP SRBC [1]	mix, labrador, other	male [18] female [22]	2.8–14.5	8–72	RBC transfusion	Association of prolonged storage of RBCs with hemolysis and cytokine response	8 [2 FRBC; 6 SRBC]	Thromb. disease [2] SIRS [2] MDOS [2] Sepsis [1]
Remy et al. [43] [2019]	USA	Human albumin + D42 RBC [9]	Human Hp + D42 RBC [9]	beagles	N/A	1.5–2.5 (18–30 months)	9–12.5	Pneumonia/ Sepsis	Interaction of CFH levels and haptoglobin	N/A	N/A
		0.9% saline [4]	Human Hp [4]								
Suffredini et al. [14] [2017]	USA	D7 RBC [18]	Iron sucrose [13] Ferumoxytol [11]	beagles	N/A	1–2	9–12.5	Pneumonia /Anemia	Comparison of fresh RBCs and potential risks of iron therapy	N/A	N/A
Solomon et al. [44] [2015]	USA	N/A	D7 RBC [6] D42 RBC [6]	beagles	N/A	1–2.2 (12–28 months)	9–12.5	Hemorrhagic shock	Adverse effects of prolonged stored RBCs	N/A	N/A
Cortes-Puch et al. [45] [2015]	USA	D7 RBC 5-10mL [6] D7 RBC 20-40mL [6] D7 RBC 60–80mL [6]	D42 RBC 5-10mL [6] D42 RBC 20-40mL [6] D42 RBC 60–80mL [6]	beagles	N/A	1–2.2 (12–28 months)	9–12.5	Pneumonia	Effects of different volumes on transfusion risks of prolonged stored RBCs	N/A	N/A
		UW D14 RBC [7] UW D21 RBC [6] UW D28 RBC [4] UW D35 RBC [3]	W D14 RBC [7] W D21 RBC [6] W D28 RBC [4] W D35 RBC [3]						Influence of storage time on the transfusion of washed vs. unwashed RBCs		
Wang et al. [46] [2014]	USA	D7 RBC S.a.0 [4] D42 RBC S.a.0 [4]	D7 RBC S.a.1 [4] D42 RBC S.a.1 [4] D7 RBC S.a.1.25 [12] D42 RBC S.a.1.25 [12] D7 RBC S.a. > 1.5 [4] D42 RBC S.a. > 1.5 [4]	beagles	N/A	1–2.2 (12–28 months)	10–15	Pneumonia	Contributing effects of the bacterial dose on transfusion risks of prolonged stored RBCs	N/A	N/A
Cortes-Puch et al. [47] [2014]	USA	UW D7 RBC [6] UW D42 RBC [6]	W D7 RBC [6] W D42 RBC [6]	beagles	N/A	1–2.2 (12–28 months)	9–12.5	Pneumonia	Effects of washing on CFH and iron levels during different storage times	N/A	N/A
Solomon et al. [48] [2013]	USA	D7 RBC [8]	D42 RBC [8]	beagles	N/A	1–2.2 (12–28 months)	10–15	Pneumonia	Viability of commercially available RBCs	N/A	N/A
		N/A	S.a.1 [8] S.a. 1.25 [8] S.a. 1.5 [4] S.a. 2.0 [4]						Bacterial dose finding		
		D7 RBC S.a.1.25 [12]	D42 RBC S.a.1.25 [12]						Influence of storage duration on mortality		
Standl et al. [49] [2003]	GER	D21 RBC [6]	HBOC-201 [6]	foxhound	N/A	20 ± 5 months	29 ± 4	Anemia	Tissue oxygenation potential of the alternative carrier HBOC-201	N/A	N/A
Standl et al. [50] [1996]	GER	D0 RBC [8]	D21 RBC [8] HBOC [8]	foxhound	male [15] female [9]	2 ± 0.5	30 ± 14	Anemia	Comparison of tissue oxygenation potential of stored RBCs, freshly donated blood and HBOC	N/A	N/A
Lucas et al. [51] [1996]	USA	RL + RBC [N/A]	FFP + RL + RBC [N/A]	N/A	male [22]	N/A	9–25	Hemorrhagic shock	Efficiency of resuscitation with FFPs in preventing coagulopathy	N/A	N/A
Ross et al. [52] [1990]	USA	Control [6]	RBC [6]	mongrel	male [6]	N/A	15	Hemorrhagic shock	Effects of severe blood loss on the kinetics of the complement system	N/A	N/A
LeBlanc and Edwards [53] [1986]	USA	WB [19]	RBC [19]	N/A	male [23] female [15]	3–14 days	0.57 ± 0.12 ^a^ 0.50 ± 0.11 ^b^	RBC transfusion	Effect of acute polycythemia on the disappearance rate of fibrinogen	N/A	N/A

^a^: whole blood control group; ^b^: polycythemia RBC group; Abbreviations: USA: United States of America; GER: Germany; AIHA: autoimmune hemolytic anemia; ITP: immune thrombocytopenia; FRBC: fresh red blood cell concentrates stored for either 9 days or less (median = 5 days); SRBC: stored red blood cell concentrates for 19–30 days (median = 26 days); Thromb. disease: Thromboembolic disease; SIRS: systemic inflammatory response syndrome; MODS: Multiple organ dysfunction syndromes; RBC: red blood cell concentrate; Hp: Haptoglobin; D7: stored for 7 days; D14: stored for 14 days; D21: stored for 21 days; D28: stored for 28 days; D35: stored for 35 days; D42: stored for 42 days; W: washed; UW: unwashed; S.a. 1: infected with 1 × 109 CFU/kg Staphylococcus aureus; S.a. 1.25: infected with 1.25 × 109 CFU/kg Staphylococcus aureus; S.a. 1.5: infected with 1.5 × 109 CFU/kg Staphylococcus aureus; S.a. 2.0: infected with 2.0 × 109 CFU/kg Staphylococcus aureus; HBOC: hemoglobin-based oxygen carriers; CFH: cell-free hemoglobin; FFP: fresh frozen plasma; RL: Ringer’s lactate solution; WB: whole blood.

### 3.3. Preparation and Storage of Erythrocytes

(A) Eight studies reported data on porcine erythrocytes preparation and storage, with a total number of 145 donor animals (Table 4). Group sizes varied immensely between the publications: a single donor animal was used in a pilot study by Biagini et al., and 60 donor animals were used by Alam et al. [24,29]. Three of the remaining four studies did not provide any information on preparation or storage [20,21,27]. Spoerke et al. only reported data on the strain of animals (Yorkshire crossbred swine) as well as on the preparation of plasma for lyophilization [28]. Overall, in most cases, whole blood collection was performed using citrate-phosphate-dextrose (CPD) as an anticoagulant followed by centrifugation and storage in sucrose-adenosine-glucose-mannitol (SAG-M) solution [15,22,25,26]. Only two studies stated to have used citrate-phosphate-dextrose-adenine-1 (CPDA-1) [23,24]. With CPD and CPDA-1 being the most commonly used anticoagulants, Buchholz et al. investigated the use of Adsol, an alternative electrolyte mixture that contains mannitol (750 mg/unit), as well as increased amounts of glucose (2.2 g/unit compared to 1.6 g/unit for CPD and 2.01 g/unit for CPDA-1), and reported no evidence of inappropriate osmotic diuresis or hyperglycemia, but showed significant hypocalcemia, arterial hypotension, and elevated blood glucose concentrations in CPDA-1 animals [30]. As summarized in Table 4, porcine red blood cell concentrates were stored at a temperature of 4 °C in all eight studies, with the exception of the studies of Biagini et al., who allowed a range of storage temperatures from 2 to 8 °C [15,22,23,24,25,26,29,30]. Storage time was reported to be 14 days in five studies by Patel et al., including the second group of 42-day-old red blood cells [15,22,23,24,25]. Two publications described the use of prepared erythrocyte concentrates immediately or within 24 h after collection [29,30]. Only one paper reported a mean storage time of 37 days [26]. Procedures to reduce storage lesions, such as supernatant removal by mechanical washing (CATS; Fresenius, Germany) or a combination of washing and rejuvenation with a solution rich in inosine (Rejuveso solution; Zimmer Biomer, USA) before application, were exclusively demonstrated by Wozinak et al. [22].

(B) In 11 publications, ovine erythrocytes were collected from a total of 175 donor animals, and 61 of these donor animals were previously infected with prions (Table S1) [31,33,34,35,36,37,38,39,40]. Two out of these 11 studies, however, did not provide any information on the number of donor sheep [32,41]. Similarly to porcine red blood cell concentrates, sucrose-adenosine-glucose-mannitol (SAG-M) was most frequently used as an additive solution [32,34,35,38]. Only Muenster et al. and Baron et al. described the use of Adsol [31,36]. With a mean of 35 to 42 days for the prolonged storage groups in five out of 11 studies, maximum storage time differed compared to the porcine studies that used 14 days of storage for the prolonged storage groups [31,32,34,35,36]. Only Vane et al. described similar storage times for their ovine studies as compared to porcine transfusion models with 8–10 days [40]. Washing procedures (COBE 2991 Cell Processing Set, TERUMO BCT, Lakewood, CO) to possibly reduce storage lesions were exclusively performed in a study by Muenster et al. [31].
(C) As shown in Table S2, only three publications provided data on the number of donor animals which came to a total of 28 dogs [49,50,52]. Ten studies reported no information in this regard but showed a clear predominance of allogenic donors [14,42,43,44,45,46,47,48,51,53]. With five to 42 days, a significantly broader range in storage time was used among dogs and thus deviated from the 14 days majorly described in group A [14,42,43,44,45,46,47,48,49,50]. In addition, the systematic analysis indicated differences in the use of phosphate-adenine-glucose-guanosine-saline-mannitol (PAGGS-M) as an additive solution by the majority of canine studies [49,50]. However, it should be noted that data were only available in three out of 13 publications [48,49,50].

For sheep and dogs, 11 combined studies reported data on a mean storage temperature of 4 °C, which is comparable to data reported for pigs [31,32,33,34,35,44,46,48,49,50,51].

### 3.4. Storage-Related Changes in Red Blood Cell Units

As shown in Table 4, two studies demonstrated that 14-day storage of porcine erythrocytes was characterized by a significant increase in cell-free hemoglobin [22,24]. Similar results were described in ovine transfusion models by Muenster et al. and Baron et al. with erythrocytes stored for a mean of 40 days (Table S1) [31,36]. While mechanical washing did not reduce but rather elevated the amount of cell-free hemoglobin released into the storage supernatant of porcine red blood cell units, significant reductions in cell-free hemoglobin concentrations were observed by Wozniak et al. in porcine rejuvenated units [22]. Similar results were reported by Muenster et al., where washing of stored ovine erythrocytes did not prevent an increase of plasma hemoglobin after transfusion in vivo [31].

In contrast, all porcine studies providing data on hematocrit and hemoglobin concentrations in red blood cell concentrates showed no significant differences after storage [22,24,25,26]. The mean hematocrit level, however, was lower in porcine units when compared to human units. This was explained by Patel and colleagues as a reflection of generally lower hematocrit concentrations in porcine venous blood (mean 26.3% ± 1.3) compared to human blood [26]. Corresponding results of hematocrit levels slightly below the required standards for units of packed red blood cells according to the Council of Europe Guidelines (48.49 ± 7.68% vs. 50–70% in humans) were demonstrated in an ovine transfusion model by Simonova et al. [34,54]

All eight studies providing information on the hemolysis index showed a significant increase in the hemolysis index with storage time among all included species (Table 4, Tables S1 and S2). The mean hemolysis index, however, remained less than one and, therefore, within the range used as a quality control parameter for human erythrocyte storage [22,24,25,26,34,35,47,48]. Data on nitric oxide bioavailability in porcine red blood cell concentrates, which is reduced as a consequence of intravascular hemolysis, was solely reported by Wozniak et al. However, the authors did not detect any significant differences between 14-day-old stored porcine red cell units that were washed, rejuvenated, or not further processed before transfusion [22].

While Biagini et al. described no significant changes in the amount of viable red blood cells during the first 24 h after transfusion of porcine erythrocytes, Wozniak et al. reported a 24 h in vivo red cell survival ranging from 68 to 72% (Table 4) [22,24]. Corresponding percentage ranges of 60 to 78.3 % after 40 and 42 days of storage were also observed in ovine and canine transfusion models (Tables S1 and S2) [31,48]. Muenster et al. reported an improvement of 24 h in vivo survival rates from 73.4% to 78.3% by nitric oxide treatment prior to transfusion in ovine models [31]. Latter values are similar to the number of viable red blood cells observed 24 h after transfusion in humans when erythrocyte concentrates at the end of storage shelf life were transfused [22].

In human erythrocytes, 2,3-diphosphoglycerate (2,3-DPG) decreases significantly within two weeks of storing and processing [5]. Similar observations were made in 14-day-old stored porcine red blood cells and in an ovine transfusion model [22,25,26,41]. Furthermore, adenosine triphosphate content decreased by 55% during storage in humans and, similarly, by 50% in porcine erythrocytes [5,22]. As demonstrated by Wozniak and colleagues, washing stored porcine erythrocytes removed microvesicles in the supernatant of stored packed red blood cells. Thereby, microvesicle-induced leukocyte activation and inflammation were attenuated [22,25,26,41]. Morphological changes in erythrocytes, such as increased membrane rigidity and the development of echinocytosis that can be associated with adenosine triphosphate depletion, were observed by two studies performed on pigs [22,25]. Muenster et al. also reported a possible improvement in the deformability of stored ovine erythrocytes by an ex vivo nitric oxide treatment prior to transfusion [31]. At the same time, extended storage decreases erythrocyte deformability and makes it hard for the red cells to transfer small capillaries; also, the rate of intravascular hemolysis after transfusion might be increased [5]. Consecutively the lifespan of transfused stored erythrocytes will be reduced [31]. With nitric oxide treatment before the transfusion of stored, packed red blood cells, erythrocyte deformability is increased [31]. Loss of erythrocyte deformability leads to increased intravascular hemolysis after transfusion and a consecutive reduced lifespan of transfused stored erythrocytes [31]. Twenty-two out of 36 studies did not report specific markers that pertain directly to changes during storage time [4,15,20,21,23,29,30,32,33,37,38,39,40,42,43,44,45,46,49,51,52,53].

## 4. Discussion

In this scoping review, a total of 36 publications providing information on erythrocyte transfusion in porcine, ovine, and canine animal models were evaluated. In seven porcine studies, blood was collected in CPDA-1 or CPD anticoagulant and stored in SAG-M solution at a temperature of 4 °C, which resembles the current practice of most European blood banks [15,22,23,24,25,26,30,55]. Corresponding values on preparation and storage temperature were also reported by the studies in sheep and dogs that were also included in this scoping review [14,31,32,33,34,35,36,37,38,40,41,44,46,48,49,50,51,53]. All publications providing data on cell-free hemoglobin levels and the hemolysis index equally described a significant increase of both parameters over storage time among all three species [22,24,25,26,31,34,35,36,47,48]. These findings correspond to similar changes observed in human red cell concentrates [4,56,57].

Storage times, however, differed with a mean of 14 days in porcine transfusion models and a mean of 35–42 days in ovine models from the mean of 22.7 days of human red cell concentrates reported by the Association for the Advancement of Blood & Biotherapies (AABB) [15,22,23,24,25,31,32,34,35,36,55,58]. Only Standl et al. and Cortes-Puch et al. reported similar storage times of 21 days in canine animal models [45,49,50].

With a majority of six studies, erythrocyte transfusion was most frequently carried out in female swine [15,22,25,26,29,30]. In contrast to porcine transfusion models, ovine and canine models were mainly dominated by male animals [34,35,42,50,51,52,53]. As shown by Tzounakas et al. in a recent study in humans, the donor’s sex affects storage lesions by impacting hemoglobin concentration, redox parameters, and membrane remodeling of stored red blood cells [59]. However, the sex-related influences on the functionality of collected erythrocytes, potentially affected by the different hormone profiles, were not addressed as confounding factors in any of the publications analyzed in this scoping review. Further donor characteristics such as age were only poorly reported in porcine transfusion models [12,13]. In contrast, sheep were most frequently aged older than three months, and the reported age of dogs ranged between 1 and 2.5 years [14,31,32,34,35,36,37,43,44,45,46,47,48,50].

In six out of eight studies, a clear predominance of allogenic blood transfusion was observed in porcine transfusion models [15,22,23,24,25,26]. A similar preference for allogenic donors was also shown in ovine and canine models [14,32,33,34,35,37,38,41,42,43,44,45,46,47,48,51,53].

Regarding the 24 h in vivo survival rate of packed red blood cells, there were conflicting results. While Wozniak et al. provided data on 24 h in vivo red cell survival in pigs with a red cell survival range of 68–72% was similar to human units, Biagini et al. described that in contrast to stored human erythrocytes, there was no significant reduction in viable porcine red blood cells 24 h after transfusion [22,24]. In addition, porcine erythrocytes demonstrate a higher degree of random destruction in comparison to human erythrocytes [60]. Therefore, the evidence on markers describing the maintenance of corpuscular integrity is very low in porcine transfusion models [3,22,24]. In concordance with the results by Wozinak et al., a reduction in viable red blood cells 24 h after transfusion was also observed in ovine and canine transfusion models [31,48]. Muenster et al., however, pointed out that the mean 24 h post-transfusion in vivo survival rate of ovine-stored erythrocytes was lower than the one reported in humans [31]. Taken together, with a total of 4 out of 36 publications providing data on the 24 h in vivo survival rate, it remains unclear whether shelf life can be adequately modeled by any of the three species [8,22,24,31].

Similar to studies performed with human red cell concentrates, a significant reduction of 2,3-diphosphoglycerate concentrations over storage time was observed in porcine units [22,25,61]. Ovine and canine transfusion models included in this scoping review reported no data in this regard but generally described lower 2,3-diphosphoglycerate levels in ovine red blood cell units compared to humans in a previously published study by Baron et al. [62]. Corresponding reductions of 2,3-diphosphoglycerate, glucose and adenosine triphosphate levels in ovine-packed red blood cells throughout storage were also reported in a recent study by Simonova et al. [63]. The depletion of adenosine triphosphate levels in porcine-packed red blood cells occurred more rapidly than those reported in human units [22,25,26]. However, adenosine triphosphate levels were restored by rejuvenation in one study [22]. Higher levels of adenosine triphosphate were also demonstrated for rejuvenated human samples by Tchir et al. [64].

Furthermore, 14-day storage of porcine erythrocytes was associated with major morphological changes such as loss of biconcave shape or elevation of membrane rigidity as described by Wozniak et al. and Patel et al. [22,25]. This alteration of shape and loss of deformability were similarly described in several studies of human-packed red blood cells [65,66,67].

The broad inclusion of synonyms for “red blood cell transfusion”, plurals and different spellings in the search strategy, as well as the application of search terms to all fields, was proven to comprehensively identify publications relevant to this work. However, the combined search term “animal model” might have limited the results of this scoping review since potentially relevant studies solely describing their models by terms such as porcine, ovine, or canine might not have been identified. In this regard, two important previously published studies that served as a reference in the included studies by Baron et al., and Remy et al. should be pointed out as they were not covered by the applied search strategy [62,68]. Alternative, broader search strategies resulted in several tens of thousands of hits, therefore not feasible for appropriate literature screening for this scoping review.

The study of Buchholz et al. and the two studies carried out by Biagini et al. used relatively small group sizes for their porcine transfusion models. Similarly, few ovine and canine studies were limited by small group sizes [34,41,44,49,52]. Therefore, statistical inaccuracies by a type II error need to be considered as a limiting factor [23,24,30]. Moreover, this review is characterized by the heterogeneity of model types as well as the high prevalence of hemorrhagic shock models among porcine models and the predominance of septic canine models (Table 1). Many studies mentioned only several aspects of blood storage-related outcome variables with a high degree of non-reported data. The high degree of heterogeneity and incomplete quantitative data provided in the included papers precluded a thorough meta-analysis from becoming impossible. The n = 138 excluded “reviews and meta-analyses” incorporated only n = 5 meta-analyses. Of those, three meta-analyses are based on clinical trials only, and one publication describes an animal model and includes a meta-analysis of clinical data in humans [69].

Also, the inclusion of comorbidities such as trauma or induced infections limits the independent assessment of storage-related effects on recipients of erythrocyte transfusions. For example, S. aureus can independently cause hemolysis [48]. However, even when using healthy swine models, it should be considered that chronic anemia is very common in farm-bred swine, particularly in sows as well as in piglets. Thus, chronic anemia might act as a potential confounding factor in porcine animal models [22,70,71]. Furthermore, the differences in porcine and human erythrocyte metabolism with regard to red blood cell storage should be taken into consideration. Patel and colleagues noted that in contrast to any other mammalian cell type, porcine red blood cells are characterized by the predominance of hexokinase III [25]. The enzyme hexokinase III accounts for 98% of the total glucose phosphorylating activity of porcine erythrocytes, affecting their ability to transport and metabolize glucose [72,73]. This, and the inhibition of relevant enzymes necessary for glycolysis, leads to a more rapid decline of adenosine triphosphate levels compared to human red cell concentrates [25,72,73]. Slower metabolic rates in mature porcine erythrocytes compared to other mammalian red blood cells were also observed in a study by Dixon et al. [74]. As pointed out in a recent publication by Simonova and colleagues, major differences in erythrocyte metabolism were also detected between ovine and human-packed red blood cells [63]. Especially maximum activity of glucose-6-phosphate dehydrogenase (G-6-PD), which serves an important role in protection against oxidative damage and glycolysis, was noticeably lower than in human erythrocytes [63,75,76]. Lower glycolytic activity and slower response to pH changes were furthermore reported in canine erythrocytes when compared with human red blood cells [77]. As reported in other publications, ovine erythrocytes are more prone to osmotic fragility due to their spherical shape and smaller size compared to human red blood cells [63,78,79]. Therefore, for the translation of improved deformability of stored ovine erythrocytes by an ex vivo nitric oxide treatment prior to transfusion, as demonstrated by Muenster et al., further tests in humans are necessary [31].

With strong similarities observed in storage conditions, this scoping review suggests high physiological comparability of porcine models with humans in regard to the red blood cell physiology during the early storage period [15,22,23,24,25,26,29,30]. However, porcine erythrocytes have a significantly shorter lifespan compared with human, ovine, and canine red cells [80]. Thus far, it is unclear which storage duration in swine would mimic the maximum allowed storage duration of human red cell concentrates of 42 days. Therefore, the transfusion-associated effects reported by the only porcine study that used red cells stored for 37 days might not be reproducible in humans [23,26]. For ovine and canine studies, there are only a few data reported on erythrocyte metabolism during early storage. Regarding the red blood cell storage lesion at the end of the storage interval, only ovine and canine studies model typical human storage for up to 42 days. Moreover, with only 4 out of all included 36 publications providing data on the 24 h in vivo survival rate of red blood cells, it remains unclear whether erythrocyte shelf life can be adequately modeled by a model of any of the three species [22,24,31,48].

For human-stored red blood cells, donor sex appears to have an impact on hemoglobin concentration and redox parameters of stored erythrocytes [59]. Furthermore, menopause seems to promote membrane remodeling of red cells, especially during prolonged storage [59]. Furthermore, osmotic fragility, an aspect that is very important for hemolysis during storage and after transfusion of stored blood, appears correlated with donor sex [81]. So far, comparable data for the impact of donor sex on the storage lesion in large animal red blood cells are poor. In addition, there were only a few studies that were selected for this review that reported the sex of the used animals. If reporting occurred and with the exception of three canine studies, only one particular sex, either female or male, was chosen for the experiments. Therefore, in this Review, no assessment of the relevance of large animal donor sex on the storage lesion could be made.

The inherent heterogeneity of included studies with vast variations in group sizes, model types, comorbidities, and strain of animal, as well as the observed differences in in vivo survival rates, erythrocyte metabolism and morphology, however, do require further research. To fully assess the value and future potential of animal transfusion models in translational research, subsequent studies should address the reported differences in erythrocyte metabolism as well as donor-related influences such as gender, the form of donation (allogenic/autologous), or pre-existing comorbidities like chronic anemia. Furthermore, prospective studies should include top-load transfusion regimens in healthy models to avoid additional potential confounding factors such as infections, hemorrhagic shock, or trauma. This would guarantee a more precise evaluation of the physiological and biochemical effects upon transfusion of stored red blood cells and might allow testing of novel pharmacological or biophysical approaches, especially in double-hit settings such as transfusion of special blood products in hemorrhagic shock. This is why in an era of in vitro single-cell test procedures or safe labeling of human erythrocytes, animal models still remain relevant for the appropriate translation of new therapeutic concepts and medications into the clinical setting [82,83].

## 5. Conclusions

Based on a natural red cell lifespan similar to that of humans, ovine and canine transfusion models can mimic typical human erythrocyte storage for up to 42 days. Porcine models stand out for reliably simulating double-hit pathologies, most importantly hemorrhagic shock. Although new concepts evolve from studying transfusion-related hypotheses directly in humans and although each large animal model and each species can only simulate certain aspects of human physiology, large animal models remain an important area of translational research since they have an impact on testing new pharmacological or biophysical interventions to attenuate storage-related adverse effects and allow, in a controlled environment, to study background and interventions in dynamic and severe disease conditions.

## Figures and Tables

**Table 4:** Preparation of porcine erythrocytes and storage lesion, sorted by year of publication.

Author [Reference] [Year]	Donor [Number]	Rate of Infusion (mL/h)	Centrif. [min]	Storage				24 h In Vivo RBC Survival (%)	Hemoglobin (g/dL) [Baseline]	Free Hemoglobin (mg/dL) [Baseline]	Hematocrit (%) [Baseline]	Hemolysis Index (%) [Baseline]	2,3-DPG conc. (μmo/g Hb) [Baseline]	ATP conc. (μmol/g Hb) [Baseline]
				Anticoagulant	Additive Solution [mL/unit]	Temp. (°C)	Time (Days)							
Wozniak et al. [22] [2018]	allogenic [11]	N/A	N/A	CPD	SAG-M	4	14	72 ^a^ 71 ^b^ 68 ^c^	14.2 ± 2.3 ^a^ 12.8 ± 1.3 ^b^ 12.4 ± 0.5 ^c^ [14.5 ± 2.45]	26 ^a^ 103 ^b^ 6 ^c^ [9]	48.8 ± 7.9 ^a^ 49.7 ± 6.5 ^b^ 42 ± 14 ^c^ [49.4 ± 8.4]	0.1 ± 0.04 ^a^ 1.0 ± 0.02 ^b^ 0.1 ± 0.03 ^c^ [0.07 ± 0.1]	6.5 ± 1.6 ^a^ 13.8 ± 0.1 ^b^ 18.3 ± 7.5 ^c^ [14.4 ± 4]	0.8 ± 0.3 ^a^ 1.3 ± 0.1 ^b^ 1.8 ± 0.4 ^c^ [2.0 ± 0.6]
Biagini et al. [23] [2018]	allogenic [16]	1785 ^†^	1130 g [16]	CPDA-1	N/A	4–8	14	N/A	N/A	N/A	N/A	N/A	N/A	N/A
Watts et al. [15] [2015]	allogenic [24]	1800 ^†^	N/A	CPD	SAG-M	4	14	N/A	N/A	N/A	N/A	N/A	N/A	N/A
Biagini et al. [24] [2014]	allogenic /autologous [1]	N/A	3300 rpm [16]	CPDA-1	N/A	2–6	14	97.5	22.2 ± 1.5 [23.3 ± 1.4]	112.4 ± 31.4 [31.0 ± 9.3]	71.1 ± 2.3 [73.3 ± 3.5]	0.5 ± 0.1 [0.1 ± 0.1]	N/A	N/A
Patel et al. [25] [2013]	allogenic [15]	250	N/A	CPD	SAG-M	4	14 vs. 42	N/A	N/A	N/A	33 ^a,^* 38 ^d,^* [36 *]	0.3 ^a,^* 0.87 ± 0.59 ^d^ [0 *]	12 ^a,^* 1 ^d,^* [15 *]	1 ^a,^* 0 ^d,^* [6 *]
Patel et al. [26] [2011]	allogenic [8]	250	2000 rpm [5]	CPD	SAG-M	4	37 (34–42)	N/A	12.1 (0.25) ** [11.7 (0.41) **]	N/A	37.2 (2.66) ** [35.7 (1.23) **]	0.87 (0.24) ** [0.01 (0.0) **]	N/A	0.01 (0.0) ** [3.24 (0.53) **]
Alam et al. [29] [2009]	autologous [60]	3000 ^†^	5000 rpm [15]	N/A	N/A	4	1	N/A	N/A	N/A	N/A	N/A	N/A	N/A
Buchholz et al. [30] [1999]	autologous [10]	999	N/A	CPD	Adsol [63] vs. CPDA-1 [100]	N/A	0	N/A	N/A	N/A	N/A	N/A	N/A	N/A

^a^: 14-day-old stored porcine red cell units; ^b^: washed 14-day-old stored porcine red cell units; ^c^: rejuvenated 14-day-old stored porcine red cell units; ^d^: 42-day-old 14-day-old stored porcine red cell units; ^†^: calculated value; *: estimated value according to published figure; **: values are least square means. Abbreviations: RBC: red blood cell; 2,3-DPG conc.: 2,3-diphosphoglycerate concentration; ATP conc.: adenosine triphosphate concentration; CPD: citrate-phosphate-dextrose solution; CPDA-1: citrate-phosphate-dextrose-adenine-1 solution; SAG-M: sucrose-adenosine-glucose-mannitol solution; Adsol: adenine, dextrose, sorbitol, sodium chloride and mannitol solution.

## Data Availability

Data are available from the corresponding author on reasonable request.

## Supplementary Materials

The following supporting information can be downloaded at: https://www.mdpi.com/article/10.3390/medicina58121735/s1, Table S1: Preparation of ovine erythrocytes and storage lesion, sorted by years of publication; Table S2: Preparation of canine erythrocytes and storage lesion, sorted by years of publication; PRISMA 2020 checklist.
