# Supplementary material for: Large Animal Models for Simulating Physiology of Transfusion of Red Cell Concentrates—A Scoping Review of The Literature
Source: Medicina (Kaunas). 2022 Nov 27;58(12):1735. doi: 10.3390/medicina58121735 (PMC9787038; doi:10.3390/medicina58121735)
Supplement: Supplementary file 1 [file medicina-58-01735-s001.zip › medicina-2035716-Supplementary Table S1.pdf]

Table S1. Preparation of ovine erythrocytes and storage lesion, sorted by year of publication

				Storage										
**Author** [reference] [year]	**Donor** [number]	**Rate of infusion** (mL/h)	**Centrif.** [minutes]	**Anticoagulant**	**Additive solution** [mL/unit]	**Temp.** (°C)	**Time** (days)	**24h in vivo RBC survival** (%)	**Hemoglobin** (g/dL) [baseline]	**Free hemoglobin** (mg/dL) [baseline]	**Hematocrit** (%) [baseline]	**Hemolysis index** (%) [baseline]	**2,3-DPG conc.** (µmo/g Hb) [baseline]	**ATP conc.** (µmol/g Hb) [baseline]
Muenster et al. [31] [2016]	autologous [29]	600	2600 g [N/A]	CPDA	Adsol	4	2<br>40	73.4 ± 3.8 [90.8 ± 4.1]<br>78.3 ± 6.3[a] [91.4 ± 1.4][a]	N/A	65.1 ± 41.64[†] [9.84 ± 3.34][†]	N/A	N/A	N/A	N/A
McDonald et al. [32] [2015]	allogenic [N/A]	N/A	5000 g [45]	CPD	SAG-M	2-6	<5<br>35 - 42	N/A	N/A	N/A	N/A	N/A	N/A	N/A
McCutcheon et al. [33] [2015]	allogenic* [7]	N/A	N/A	CPDA-1	N/A	4	1	N/A	N/A	N/A	N/A	N/A	N/A	N/A
Simonova et al. [34] [2014]	allogenic [12]	N/A	5000 g [45]	CPD	SAG-M	2-6	<5<br>35 - 42	N/A	N/A [15.88 ± 1.54]	N/A	N/A [48.49 ± 7.68]	0.7*** [0]***	N/A	N/A
Fung et al. [35] [2013]	allogenic [14]	N/A	5000 g [45]	N/A	SAG-M	4	<5<br>35 - 42	N/A	N/A	N/A	N/A	0.2 [± 0.18][b]<br>0.81 [± 0.29][c]<br>[0]	N/A	N/A

Baron et al. [36] [2013]	autologous [17]	N/A	N/A	CPDA	Adsol	N/A	39 [± 2]	N/A	N/A	29 ± 10 47 ± 35[d] [7 ± 2]	N/A	N/A	N/A	N/A
Lacroux et al. [37] [2012]	allogenic** [15]	343[†]	3640 rpm [12]	CD	N/A	N/A	5	N/A	N/A	N/A	N/A	N/A	N/A	N/A
McCutcheon et al. [38] [2011]	allogenic [10[e] + 39[f]]	N/A	1350 [7.5]	CPDA-1	SAG-M	N/A	N/A	N/A	N/A	N/A	N/A	N/A	N/A	N/A
Jonker et al. [39] [2011]	autologous [13]	N/A	N/A	N/A	N/A	N/A	N/A	N/A	N/A	N/A	N/A	N/A	N/A	N/A
Vane et al. [40] [2002]	autologous [19]	N/A	N/A	CPDA	N/A	N/A	8 - 10	N/A	N/A	N/A	N/A	N/A	N/A	N/A
Widness et al. [41] [2000]	allogenic [N/A]	15.74[†]	N/A	CPDA	N/A	N/A	<7	N/A	N/A	N/A	N/A	N/A	8*** [28]***	N/A

a: treated with nitric oxide; b: stored for 28 days; c: stored for 42 days; d: inhaled nitric oxide; e: healthy donors; f: infected donors; *: infected with 5g of brain homogenate prepared from BSE-infected cattle; **: infected with PG127 scrapie isolate; ***: estimated value according to published figure; †: value converted from μM to mg/dl Abbreviations: RBC: red blood cell; 2,3-DPG Conc.: 2,3-diphosphoglycerate concentration; ATP conc.: adenosine triphosphate concentration; CD: citrate-dextrose solution; CPD: citrate-phosphate-dextrose solution; CPDA: citrate-phosphate-dextrose-adenine solution; CPDA-1: citrate-phosphate-dextrose-adenine-1 solution; SAG-M: sucrose-adenosine-glucose-mannitol solution; Adsol: adenine, dextrose, sorbitol, sodium chloride and mannitol solution.
