# Supplementary material for: Large Animal Models for Simulating Physiology of Transfusion of Red Cell Concentrates—A Scoping Review of The Literature
Source: Medicina (Kaunas). 2022 Nov 27;58(12):1735. doi: 10.3390/medicina58121735 (PMC9787038; doi:10.3390/medicina58121735)
Supplement: Supplementary file 1 [file medicina-58-01735-s001.zip › medicina-2035716-Supplementary Table S2.pdf]

Table S2. Preparation of canine erythrocytes and storage lesion, sorted by year of publication

**Author** [reference] [year]	**Donor** [number]	**Rate of infusion** (mL/kg/30min)	**Centrif.** [minutes]	Storage: **Anticoagulant**	Storage: **Additive solution** [mL/unit]	Storage: **Temp.** (°C)	Storage: **Time** (days)	**24h in vivo RBC survival** (%) [baseline]	**Hemoglobin** (g/dL) [baseline]	**Free hemoglobin** (mg/dL) [baseline]	**Hematocrit** (%) [baseline]	**Hemolysis index** (%) [baseline]	**2,3-DPG conc.** (µmo/g Hb) [baseline]	**ATP conc.** (µmol/g Hb) [baseline]
Callan et al. [42] [2021]	allogenic [N/A]	2.5***	N/A	N/A	N/A	N/A	5*<br>26*	N/A	N/A	N/A	N/A	N/A	N/A	N/A
Remy et al. [43] [2019]	allogenic [N/A]	20	N/A	N/A	N/A	N/A	42	N/A	N/A	N/A	N/A	N/A	N/A	N/A
Suffredini et al. [14] [2017]	allogenic [N/A]	20	N/A	CPDA-1	N/A	N/A	7	N/A	N/A	N/A	N/A	N/A	N/A	N/A
Solomon et al. [44] [2015]	allogenic [N/A]	55	N/A	CPDA-1	N/A	1-6	42<br>7	N/A	N/A	N/A	N/A	N/A	N/A	N/A
Cortes-Puch et al. [45] [2015]	allogenic [N/A]	5-80[a]	N/A	N/A	N/A	N/A	42<br>35<br>28<br>21<br>14<br>7	N/A	N/A	N/A	N/A	N/A	N/A	N/A
Wang et al. [46] [2014]	allogenic [N/A]	10	N/A	N/A	N/A	1-6	42<br>7	N/A	N/A	N/A	N/A	N/A	N/A	N/A
Cortes-Puch et al. [47] [2014]	allogenic [N/A]	13.4***	N/A	N/A	N/A	N/A	42<br>7	N/A	18.8**<br>[19]**	124.12[†]<br>[31.03][†]	N/A	0.4**<br>[0.3]**	N/A	N/A
Solomon et al. [48] [2013]	allogenic [N/A]	10	4000 rpm [6.5]	CP2D	AS-3	1-6	42<br>7	60-63<br>[69-99]	N/A	N/A	N/A	0.21**<br>[0.02]**	N/A	N/A

Standl et al. [49] [2003]	autologous [6]	N/A	N/A	N/A	PAGGS-M	4	21	N/A	N/A	N/A	N/A	N/A	N/A	N/A
Standl et al. [50] [1996]	autologous [16]	N/A	4000g [15]	CPD	PAGGS-M	4	21 0	N/A	N/A [29.1 ± 1.22]	N/A	N/A	N/A	N/A	N/A
Lucas et al. [51] [1996]	allogenic [N/A]	N/A	N/A	N/A	N/A	4	N/A	N/A	N/A	N/A	N/A	N/A	N/A	N/A
Ross et al. [52] [1990]	autologous [6]	N/A	N/A	N/A	N/A	N/A	N/A	N/A	N/A	N/A	N/A	N/A	N/A	N/A
LeBlanc and Edwards [53] [1986]	allogenic [N/A]	N/A	N/A	CPDA	N/A	N/A	N/A	N/A	N/A	N/A	N/A	N/A	N/A	N/A

*: median value; **: estimated value according to published figure; ***: calculated value; †: estimated value according to published figure converted from μM to mg/dl; a: weight-based infusion rate without a specific period of time **Abbreviations:** RBC: red blood cell; 2,3-DPG Conc.: 2,3-diphosphoglycerate concentration; ATP conc.: adenosine triphosphate concentration; CPD: citrate-phosphate-dextrose solution; CPDA: citrate-phosphate-dextrose-adenine solution; CPDA-1: citrate-phosphate-dextrose-adenine-1 solution; CP2D: citrate-phosphate-double-dextrose; AS-3: additive solution 3; PAGGS-M: phosphate-adenine-glucose-guanosine-saline-mannitol solution.
